# Efficacy of platelet-rich fibrin in promoting the healing of extraction sockets: a systematic review

**DOI:** 10.1186/s40729-021-00393-0

**Published:** 2021-12-19

**Authors:** Sarah Al-Maawi, Kathrin Becker, Frank Schwarz, Robert Sader, Shahram Ghanaati

**Affiliations:** 1 FORM, Frankfurt Oral Regenerative Medicine, Clinic for Maxillofacial and Plastic Surgery, Goethe University, Theodor-Stern-Kai 7, 60596 Frankfurt/ Main, Germany; 2 Department of Oral Surgery and Implantology, Carolinum, Goethe University, Frankfurt, Germany; 3 Department of Orthodontics, University of Düsseldorf, 40225 Düsseldorf, Germany

**Keywords:** PRF, Platelet-rich fibrin, Socket preservation, Ridge preservation, Socket healing, Pain management, Soft tissue healing

## Abstract

**Purpose:**

To address the focused question: in patients with freshly extracted teeth, what is the efficacy of platelet-rich fibrin (PRF) in the prevention of pain and the regeneration of soft tissue and bone compared to the respective control without PRF treatment?

**Methods:**

After an electronic data search in PubMed database, the Web of Knowledge of Thomson Reuters and hand search in the relevant journals, a total of 20 randomized and/or controlled studies were included.

**Results:**

66.6% of the studies showed that PRF significantly reduced the postoperative pain, especially in the first 1–3 days after tooth extraction. Soft tissue healing was significantly improved in the group of PRF compared to the spontaneous wound healing after 1 week (75% of the evaluated studies). Dimensional bone loss was significantly lower in the PRF group compared to the spontaneous wound healing after 8–15 weeks but not after 6 months. Socket fill was in 85% of the studies significantly higher in the PRF group compared to the spontaneous wound healing.

**Conclusions:**

Based on the analyzed studies, PRF is most effective in the early healing period of 2–3 months after tooth extraction. A longer healing period may not provide any benefits. The currently available data do not allow any statement regarding the long-term implant success in sockets treated with PRF or its combination with biomaterials. Due to the heterogeneity of the evaluated data no meta-analysis was performed.

## Introduction

Dental implants have become an integral part of the oral and maxillofacial surgery. They provide the most comfortable and favorable method to replace lost teeth and reconstruct the esthetic and function for the patients [1, 2]. To achieve long-term success of dental implants many clinical, biomechanical and biological requirements are needed [3, 4]. Especially, healthy and active bone and soft tissue are needed to support osseointegration. Therefore, a thorough understanding of the mechanisms of socket healing became a central research topic in the last decades [5, 6]. After tooth loss, the alveolar bone undergoes a remodeling process resulting in loss of bone quantity and changes of bone quality [7]. These processes finally lead to alveolar bone atrophy. The process of atrophy was described as a rapid and continuous process. In this context, 50–60% of the alveolar bone atrophies in the first three months after tooth extraction [6, 8]. These findings highlight the importance of the initial period after tooth extraction as critical for the further healing and changing of the alveolar bone. Accordingly, different protocols were established to avoid bone atrophy and achieve dental implantation.

Socket preservation is a prophylactic intervention that includes applying bone substitute materials (BSMs) into the extraction socket to preserve the alveolar bone dimension [9, 10]. Similarly ridge preservation is applied when tooth extraction results in a larger defect. A wide range of BSMs including synthetic and naturally derived biomaterials is available for clinical application [11, 12]. After BSMs application, a healing period of 3–6 months is recommended according to the defect morphology and the applied BSM [13–15]. During the healing period of 3–6 months, the processes of natural alveolar healing interferes with the BSM-based new bone formation and leads to the regeneration of a sufficient implant bed, that allows the delayed insertion of dental implant [16]. This two-stage implantation concept is based on the preparation of the alveolar bone prior to implant insertion. Many clinical studies reported about socket and ridge preservation using different types of BSMs [17]. However, there is still no clear evidence about the most suitable time of implant placement [18] Immediate implant placement after tooth extraction has been considered an alternative option to limit alveolar bone resorption [18, 19]. However, this approach is limited to specific socket morphologies and indications, when a sufficient bone volume is available and the buccal bone is preserved. This method can be also applied in combination with BSMs to fill the socket when needed [20].

In addition to BSMs, blood concentrate systems gained increasing importance in different fields of regenerative medicine in the last decade [21]. Blood concentrates are obtained from patients own peripheral blood [22]. Thereby, the blood components such as leukocytes, platelets, plasma proteins and growth factors are concentrated by centrifugation and prepared using different protocols [23–25]. Platelet-rich plasma (PRP) is the first generation of blood concentrates. PRP includes mainly platelets, whereas leukocytes are removed during the preparation process [25, 26]. For its preparation, the patients’ blood is centrifuged in two centrifugation steps [27]. In addition, plasma rich in growth factors (PRGF) is a further concept that utilizes the advantages of blood-derived growth factors [28]. Both systems apply a rather high relative centrifugal force (RCF) during their preparation [25]. By contrast, the second generation of blood concentrates, i.e., platelet-rich fibrin (PRF), is prepared by a one-step centrifugation without the application of any anticoagulants [29]. PRF consists of platelets, leukocytes and their subgroups embedded in a fibrin matrix with plasma proteins [21]. The first protocol of PRF applies a comparably lower, but still high RCF (≈710×*g*) [30]. This protocol was called leukocytes-rich platelet-rich fibrin (L-PRF), mainly because it contains more leukocytes compared to the first-generation blood concentrates PRP.

PRF matrices have been used in different indications in oral and maxillofacial surgery and implant dentistry [31]. Some clinical studies reported on the benefits and drawbacks of the different blood concentrate systems [32]. Recently, different systematic reviews aimed to summarize the available evidence on the use of PRF [31, 33, 34]. However, they were not focused on socket preservation, but extended their investigation to a wider range of indications and included different evidence levels. Therefore, the present systematic review aimed to focus on the role of PRF in ridge preservation to addressed the following focused questions: in patients with freshly extracted teeth, what is the efficacy of PRF in the prevention of pain and the regeneration of soft tissue and bone compared to the respective control without PRF treatment?

## Methods

This systematic review was designed and performed following the preferred reporting items of the PRISMA statement [35, 36].

### Focused question

This systematic review followed the structure of the focused questions (PICO) for the literature search [37]:

- Population (P): patients with freshly extracted teeth.
- Intervention (I): socket or ridge preservation using platelet-rich fibrin (PRF) with or without biomaterials.
- Comparison (C): spontaneous healing, biomaterials without PRF.
- Outcomes (O): measurements of at least one of the following parameters: postoperative inflammation and pain, soft tissue healing, dimensional bone volume changes, bone quality.

### Search strategy

An electronic search was conducted through PubMed and Web of Science, followed by a hand search for relevant articles published between 1990 up to June 2021. A commercially available software program (Microsoft Excel) was used for data management. Two authors (S.A. and S.G.) independently screened the identified articles. In case of disagreement regarding inclusion, detailed review of the defined criteria was performed and the disagreements were resolved upon discussion.

The combination of following keywords:

“PRF”, “platelet rich fibrin”, “socket preservation”, “ridge preservation”, “molar”, “premolar”.

Keywords combination:

("platelet rich fibrin"[MeSH Terms] OR ("platelet-rich"[All Fields] AND "fibrin"[All Fields]) OR "platelet-rich fibrin"[All Fields] OR "PRF"[All Fields] OR ("platelet"[All Fields] AND "rich"[All Fields] AND "fibrin"[All Fields]) OR "platelet rich fibrin"[All Fields]) AND ("socket" or "ridge" or "molar" or "premolar"[All Fields]).

A manual search was additionally performed in the following journals:

- International Journal of Oral and Maxillofacial Implants;
- Clinical Implant Dentistry and Related Research;
- Clinical Oral Implants Research;
- Journal of Clinical Oral Investigations
- Journal of Implantology;
- Journal of Oral and Maxillofacial Surgery;
- International journal of oral and maxillofacial surgery.

### Inclusion criteria

- English language
- Patient age 15–99 years
- Prospective controlled (CCTs) and/or randomized clinical studies (RCTs) in humans with either a split-mouth or parallel design with reasonable controls*
- Treatment of fresh sockets/ridge
- Treatment using either PRF (with or without biomaterials, i.e., bone substitute materials, collagen membranes as well as any other membrane of different origin) or spontaneous healing
- Treatment without any additional chemical or physical agents in/on the alveolus after extraction except suture materials
- Subject with and without anticoagulation intake.

### Exclusion criteria

- Preclinical in vitro or animal studies;
- Third molar extraction;
- Combination with biomaterials without reasonable controls;
- Prospective randomized and/or controlled clinical studies (RCTs) in humans with either a split-mouth or parallel design without reasonable controls;
- Case reports, case series, cohort and retrospective studies;
- Immediate implantation;
- Inadequate methods or reporting of the study design and/or patients’ data.

*reasonable controls were considered as control groups in which all applied procedures were equivalent to the test group except for PRF. Therefore, in case of the sole use of PRF in the test group, the reasonable control was considered as the spontaneous healing. In the case of the use of biomaterials in combination with PRF in the test group, the reasonable control was considered to be the application of the exact same biomaterial without PRF.

### Quality evaluation of included studies

The quality of selected RCTs was reviewed to assess the bias risk. Evaluation was performed according to the Cochrane Handbook for Systematic Reviews of Interventions version 6.2 (updated February 2021), (low, high, unclear). CCTs were evaluated according to Newcastle–Ottawa Quality Assessment Scale for non-randomized studies. The following categories were analyzed: random sequence generation, allocation concealment, blinding of participants and personnel, blinding of outcome assessment, and incomplete outcome data [38]. The assessment was conducted by two independent reviewers (SA, SG) based on the published full text article. Disagreements were resolved upon discussion.

### Data extraction

Data extraction was organized in a data-sheet including, study design, number of treated subjects, case definition, population, surgical extraction protocol, socket specifications, PRF-preparation protocol, treated groups comparison. For data analysis following parameters were defined:

- Primary outcomes: radiological and clinical evaluation of bone regeneration, dimensional bone-level change and histological assessment of bone regeneration.
- Secondary outcomes: healing period, pain management outcome and soft tissue regeneration.

## Results

### Study inclusion

**Fig. 1:**
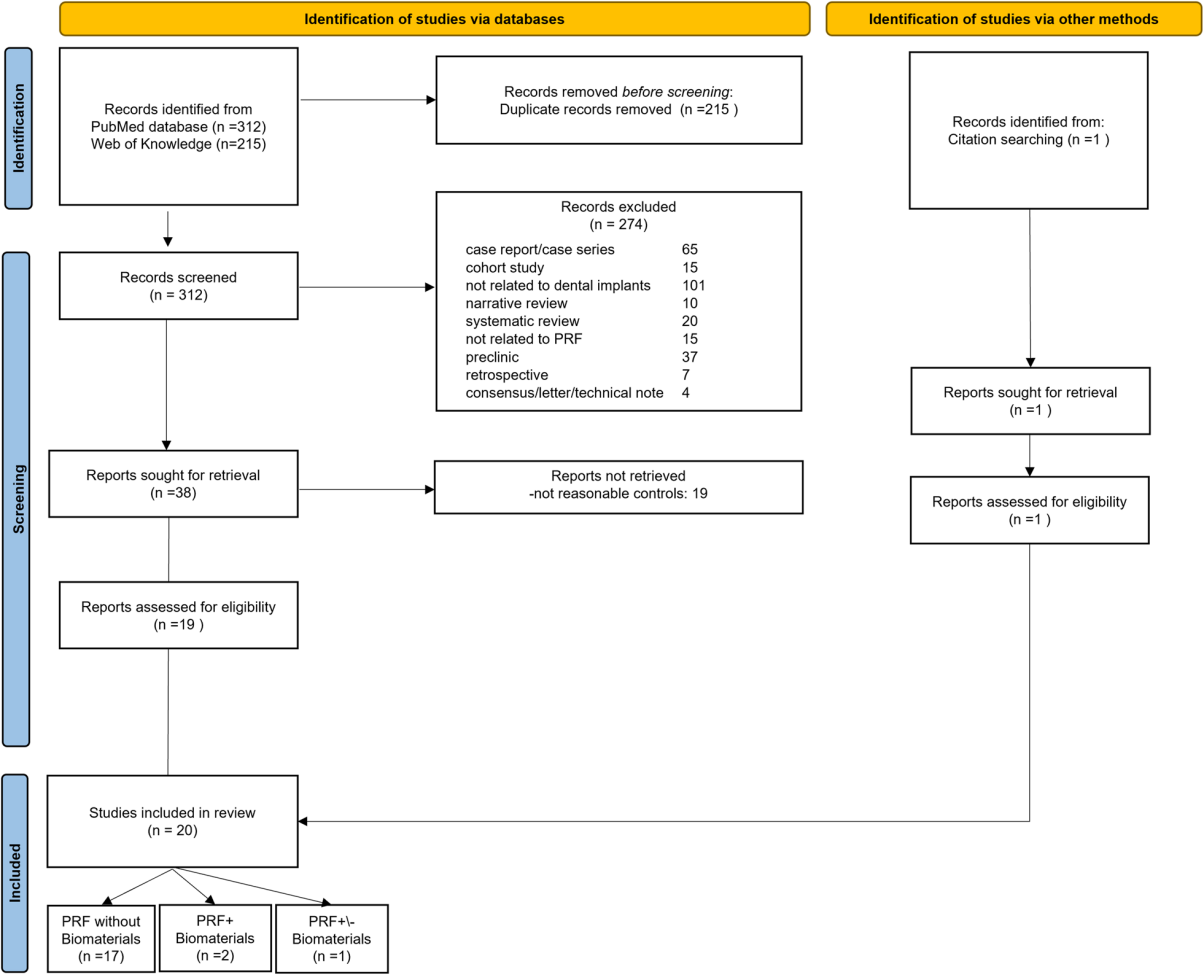
Flowchart describing the research strategy and study selection (modified according to the PRISMA 2020 statement: an updated guideline for reporting systematic reviews [36])

The PubMed and Web of Science search resulted in 312 and 215, respectively. The manual search in the relevant journals did not result in additional titles. One article was retrieved from other sources (published reviews). After removal of 215 duplicated articles, 312 titles and abstracts were reviewed from which 292 studies were excluded according to the exclusion criteria. Thirty-three full-text articles were reviewed, of which 20 were included in the qualitative analysis. Due to parameter variation and data limitation, no meta-analysis could be conducted (Fig. 1).

### Study designs

Twenty studies were analyzed in this review. Nine of the included studies were designed as parallel RCTs and seven were designed as split mouth RCT. Further two CCTs (one split mouth and one parallel study) were included in this review. One study was not further defined by the authors and one further study included both split mouth and parallel design according to the teeth needed to extract in each patient. Seventeen studies compared only the treatment of PRF as a test group to the spontaneous healing without any further treatment. One study included four groups and evaluated first the treatment of PRF alone in comparison to the spontaneous wound healing and second the combination of PRF with a bone substitute material in comparison to the bone substitute material alone [39]. Two further studies compared Bone substitute materials in combination with PRF to Bone substitute materials without PRF.

The case definition differed in the respective studies. Mainly patients in need of tooth extraction with or without dental implantation were studied. Additionally, some studies focused on single rooted teeth or premolars only. Most of the studies did not report or specify the morphology of the treated sockets/ridge. When reported, the studies included sockets with presence of 50% or more of the lingual/buccal socket walls. Most of the studies reported atraumatic tooth extraction without flap mobilization or intention of primary healing (Table 1).

**Table 1:** Study design

Study	Design	Number of patients	Test (*n*)	Control (*n*)	Manage	Case definition	Socket definition	Surgical procedure	Region/tooth	Healing time until implantation
Castro et al. [41]	Split mouth RCT	21 (15 females, 6 males)	21	21	Not reported	Patients in need of at least three tooth extractions in the aesthetic zone were	Not reported	Tooth extractions were performed under local anesthesia and sterile conditions with a flapless approach	Premaxilla	3 months
Sharma et al. [54]	Split mouth RCT	30 (16 females and 14 males)	30	30	23.90	Patients requiring extraction of bilateral mandibular molars except third molars	Not reported	Extraction of the tooth on both case and control side was done as atraumatic as possible in the same appointment	Not reported	Not reported
Mourao et al. [56]	Parallel RCT	32 (19 females and 13 males)	16	16	37	Patients requiring posterior tooth extraction (third molar exception) in the mandible or maxilla region were included	Not reported	All teeth were extracted using a minimally traumatic procedure. No vertical releasing incisions were performed. To avoid root and bony fractures, the molar teeth were sectioned using a multilaminated drill. Luxation of the teeth was performed using a periotome followed by removal using forceps	Molars and premolars	Not reported
Canellas et al. [47]	Parallel RCT	48 (27 females, 21 males)	24	24	44.8	Patients in good general health requiring a single, non-molar tooth extraction	Presence of buccal and palatal/lingual bone walls	Teeth were extracted using small levers without any osteotomy or mucoperiosteal flap, to minimize trauma	Incisors, canines and premolars	3 months
Srinivas et al. [48]	CCT split moth	30 (not further specified)	Not reported	Not reported	Not reported	Healthy subjects with chronic periodontal conditions and who had teeth indicated for extractions	Not reported	Extraction of teeth was performed with emphasis on atraumatic extraction methods. A periosteal elevator was used to reflect the gingival tissues surrounding the tooth. Tooth was luxated from its socket using periotomes and/or luxators. Appropriate forceps were used depending on the availability of tooth structure to complete the extraction process in the maxilla or the mandible	Maxilla and mandible	Not reported
Ahmed et al. [53]	Parallel RCT	54 (22 females, 32 males)	Test 1:18, test 2: 18	18	Not reported	Patients requiring extractions of maxillary or mandibular teeth and who desire replacement of teeth by dental implants	Not reported	Not reported	Not reported	Not reported
Areewong et al. [55]	Parallel RCT	36 (21 females and 15 males)	18	18	50.67	Healthy volunteers above 20 year of age, no systemic pathoses, that could disturb implant placement; Single rooted premolars and/or maxillary anterior teeth with an indication to extract and to be replaced with a dental implant	Intact surrounding alveolar bone (remaining bone at least two-third of root length)	A minimally traumatic extraction technique was performed. The periodontal ligaments were gently cut with a Piezotome. The tooth was carefully mobilized using forceps without flap reflection	Single rooted premolars and/or maxillary anterior teeth	2 months
Ustaoglu et al. [42]	Parallel RCT	57 (29 females and 28 males)	Test1:19 Test2: 19	19	Female: 35.9 Male:37.91	Patients in need of single-rooted tooth extraction with the persistence of 50% or more of bone support (anterior or premolar teeth); demanded a single implant-supported prosthetic restoration in a premolar or anterior site	Persistence of 50% or more of bone support	The tooth was extracted using a flapless technique with as little trauma to the bone and soft tissue	Single-rooted tooth	Not reported
Giudice et al. [40]	Split mouth RCT	40 (12 females and 28 males)	40	40	60.9	Patient taking long-term oral antiplatelets and requiring at least four extractions of non-adjacent teeth	Not specified	Teeth extractions were performed as atraumatically as possible attempting to preserve the alveolar bone. Molars were sectioned with drills in two or three parts. Extraction sockets were carefully cleaned from any remains of granulation tissue. Flapless extractions were attempted, but if necessary flaps were elevated at the discretion of the Operator	All regions	Not reported
Zhang et al. [49]	Parallel CCT	28 (14 females and 14 males)	14	14	34.6	Patients with upper and lower mandibular molars diagnosed as fractured tooth or could not be retained for other reasons	Not reported	All patients were treated with the non‑flap minimally invasive extraction technology	Molars	3 months
Kumar et al. [43]	RCT Parallel/Split	48 (not further specified)	Not reported	Not reported	44.4	Patients requiring tooth extraction	Not reported	All teeth were extracted atraumatically using periotomes and luxators without raising mucoperiosteal flap	All regions	Not reported
Asmael et al. [58]	RCT split mouth	20 males	20	20	44.2	Smoker patient with multiple teeth extraction	Not reported	Extraction in atraumatic manner	All regions	Not reported
Clark et al. [39]	Parallel RCT	45 enrolled 40 analyzed (22 females and 18 males)	Test 1:10 Test2:10	Control1:10 Control2:10	58	Patients with single-rooted tooth requiring extraction and replacement with a dental implant supported restoration	Teeth were excluded if they demonstrated a buccal dehiscence of more than 25% of the length of the tooth or presence of acute infection of endodontic origin	Non-traumatic tooth extraction was completed without the elevation of a mucoperiosteal flap	Not reported	3.75 months
Alzahrani et al. [44]	Parallel RCT	24 (15 females and 9 males)	12	12	37.8	Subjects with at least one site bordered by minimum of one tooth, nonsmokers, teeth with root fracture, patients having teeth with hopeless periodontal prognosis, teeth with failed endodontic therapy or advanced carious lesion	Not reported	The teeth were extracted with minimal trauma and without flap elevation, using periotomes by single experienced periodontist	Not reported	Not reported
Temmerman et al. [50]	Split moth RCT	22 (15 females and 7 males)	22	22	54	Symmetrical bilateral (e.g., premolar versus premolar, incisor versus incisor) tooth extractions in the maxilla or mandible	Not specified	A flapless approach, as atraumatically as possible using periotomes, was used. Sites with loss of the buccal or palatal bone plate (< 50% of the initial height) were not excluded. Sockets were carefully cleaned using curettes	Incisors, canines and premolars	Not reported
Marenzi et al. [57]	Split mouth RCT	26 (17 females and 9 males)			53	Patients who needed bilateral paired dental extractions	All extraction sites were simple with alveolar walls preserved	The teeth were extracted in a nontraumatic manner without elevation of full-thickness flaps and preserving the buccal and lingual walls of the sockets	Canines, premolars and molars	Not reported
Suttapreyasri et al. [45]	Split mouth RCT	8 (5 women and 3 men)	8	8	20.3	Patients who are physically healthy, with no underlying systemic disease, as determined by medical history records. Need for symmetrical premolars extraction	Not reported	The tooth was gently luxated with an elevator and carefully extracted with extraction forceps, attempting to minimize the trauma to the bone circumscribing the alveolus	Premolars	Not reported
Hauser et al. [46]	Parallel RCT	23 (9 females and 14 males)	Test 1: 19 Test 2: 6	8	47.43	Patients, who required the extraction of an upper or lower premolar before its replacement by a dental implant	Presence of the buccal and palatal/lingual bony walls evaluated clinically by measuring the thickness of the alveolar ridge and radiologically by a periapical radiograph, and residual periodontal attachment of at least 6 mm	A scalpel for the syndesmotomy; a buccal and palatal/lingual mucosal flap without discharge for the PRF-flap group; use of dental elevators, extraction with forceps, curettage, and socket filling with PRF membranes for PRF and PRF-flap groups; placement of the PRF membranes over the alveolar crest for the PRF-flap group; hemostasis by compression; and suture with a point cross	Premolars	2 months
Thakkar et al. [52]	RCT (not further defined)	36 sites (Number of patients not defined)	Not reported	Not reported	Not reported	Patients between the age group of 20 and 55 years, requiring extraction of at least one maxillary or mandibular nonrestorable single‑rooted tooth	Not described	Periotomes and forceps were used with great care taken to maintain the buccal bone and the surrounding soft and hard tissues	Single rooted teeth	Not reported
Yewale et al. [51]	Parallel RCT	20 (9 females and 11 males)	Not reported	Not	35	Sites in maxilla were selected which where, single non restorable teeth and indicated for extraction	Intactness of buccal cortical plate was examined and assessed	Extraction was carried out atraumatically. Subsequent to atraumatic extraction, height of buccal and palatal bone plate was clinically inspected at mid buccal and mid lingual region with aid of periodontal probe. With #15 blade, intrasulcular incision was made elevating marginal gingiva and adjacent interdental papilla. Flap reflection was done by Periosteal elevator resulting in exposure of crestal bone around socket. This aided in direct visualization and measurement of crestal bone level. Bone curette was used to debride extraction socket if granulation tissue is present	Single rooted teeth	Not reported

### Evaluation of bias risk

**Fig. 2:**
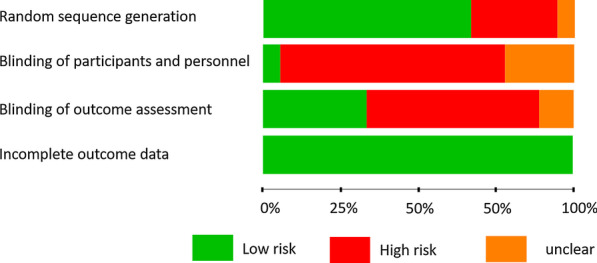
Bias risk assessment of RCTs according to the Cochrane collaborations tool

The reviewer judgment on the bias risk of RCTs showed that the highest bias risk was assessed in the categories blinding of participant and personnel as well as blinding of outcome assessment (Table 2, Fig. 2). The highest bias risk of the two included CCTs was referred to case selection and comparability (Table 3).

**Table 2:** Risk bias assessment according to according to the Cochrane collaborations tool

Study	Random sequence generation	Allocation concealment	Blinding of participants and personnel	Blinding of outcome assessment	Incomplete outcome data
Castro et al. [41]	+	n.a	−	+	+
Sharma et al. [54]	+	n.a	−	−	+
Mourao et al. [56]	+	n.a	−	−	+
Canellas et al. [47]	+	n.a	+	+	+
Ahmed et al. [53]	−	n.a	−	−	+
Areewong et al. [55]	+	n.a	−	−	+
Ustaoglu et al. [42]	+	n.a	Participant - Personnel +	+	+
Giudice et al. [40]	+	n.a	–	+	+
Kumar et al. [43]	−	n.a	?	−	+
Asmael et al. [58]	−	n.a	−	−	−
Clark et al. [39]	+	n.a	?	+	+
Alzahrani et al. [44]	−	n.a	−	−	+
Temmerman al. [50]	+	n.a	−	−	+
Marenzi et al. [57]	+	n.a	?	?	+
Suttapreyasri et al. [45]	−	n.a	−	−	+
Hauser et al. [46]	?	n.a	−	?	+
Thakkar et al. [52]	+	n.a	−	−	+
Yewale et al. [51]	+	n.a	−	+	+

+ low ristk, −high risk, ? unclear risk, n.a., not applicable

**Table 3:** Risk bias assessment according to Newcastle–Ottawa Quality Assessment Scale case–control studies

	Selection	Comparability	Exposure
Srinivas et al. [48]			
Zhang et al. [49]			

### PRF preparation protocol

Most of the included studies evaluated the L-PRF protocol. Additionally, Giudice et al. [40] and Castro et al. [41] evaluated A-PRF + in comparison to L-PRF, Clark et al. [39] evaluated A-PRF and Ustaoglu et al. [42] analyzed T-PRF in comparison to L-PRF. Most of the studies reported solely the used rounds per minutes (rpm) and centrifugation time without referring to the centrifuge design or the applied relative centrifugal force (Table 4).

**Table 4:** Centrifugation protocols used in each study

Study	PRF-type	Tube	RPM (RCF [×g])	Centrifugation time (min)	Centrifuge
Castro et al. [41]	L-PRF	9­ml silica-­coated plastic tubes without anticoagulant (BVBCTP-­2, Intra-­Spin, Intra-­Lock)	2700 rpm (RCF_clot_: 408 g)	12	Intra-­Spin, Intra-­Lock
	A-PRF +	10-ml glass tubes without anticoagulant (DUO) for A-­PRF +	1300 rpm (RCF_clot_: 145 g)	8	DUO Process
Sharma et al. [54]	PRF	6 ml intravenous blood was collected in a 10-ml sterile tube without anticoagulant	3000 rpm	10	LabTech AVI-532-BL centrifugation machine
Mourao et al. [56]	L-PRF	10-ml red tubes (IntraSpin™, Biohorizons®)	2700 rpm (708×*g*)	12	IntraSpin™, Biohorizons®, Birmingham, Alabama, USA
Canellas et al. [47]	L-PRF	sterile, glass-coated plastic tubes	2700 (708×*g*)	12	Intra-Lock, Boca Raton, Florida, USA
Srinivas et al. [48]	L-PRF	10 ml test tubes which were kept without an anticoagulant	3000 rpm	10	Not reported
Ahmed et al. [53]	L-PRF	Not reported	3000 rpm	10	Not reported
Areewong et al. [55]	L-PRF	Glass tube	2700 rpm	12	IntraSpin, Intra-Lock, Nice, France
Ustaoglu et al. [42]	L-PRF	9 mL tubes	2700 rpm	12	Intra-Spin System, L-PRF kit, Intra-Lock, Boca-Raton, FL, USA
	T-PRF	Grade IV sterile titanium tubes	2800 rpm	12	Not reported
Giudice et al. [40]	A-PRF +	A-PRF + tubes	1300 rpm	8	DUO centrifuge (Process for PRF, Nice, France);
	L-PRF	Red tubes	2700 rpm	18	(Intra-Lock International, Boca Raton, Florida, USA
Zhang et al. [49]	L-PRF	test tubes without any anticoagulant	400×*g*	10	Hettich® Universal 320 (Andreas Hettich GmbH & Co.KG, Tuttlingen, Germany)
Kumar et al. [43]	PRF	Not reported	3000 rpm	10	Not reported
Asmael et al. [58]	PRF	Five or ten milliliters of intravenous blood was drawn in 10 mL glass vacuumed tube without anticoagulants	3000 rpm	10	Centrifuge machine (Xiangtian, Jiangsu China)
Clark et al. [39]	A-PRF	01 mL sterile glass vacuum tube	1300 rpm	8	Not reported
Alzahrani et al. [44]	PRF	Not reported	3000 (400×*g*)	10	Compact centrifuge (Hermle labortechnik, Germany)
Temmerman et al. [50]	L-PRF	Plastic 10-mL tubes without anticoagulant	2700 rpm	12	(IntraSpin™, IntraLock, Boca Raton, Florida, USA)
Marenzi et al. [57]	L-PRF	9-mL tubes	2700 rpm	12	Intra-Lock, Boca-Raton, FL, USA
Suttapreyasri et al. [45]	L-PRF	10-mL glass tube	3000 rpm	10	Hettich Zentrifugen centrifuge EBA 20 (Andreas Hettich GmbH& Co, KG, Tuttlingen, Germany
Hauser et al. [46]	PRF	8-mL tubes without anticoagulant	2700 rpm	8	Not reported
Thakkar et al. [52]	PRF	10 ml syringe	3000 rpm	10	Not reported
Yewale et al. [51]	A-PRF +	10-mL tubes without anticoagulants	1300 rpm (208×*g*)	8	Not reported

### Primary outcomes

The results of the primary outcomes are described below.

#### Bone regeneration

Fifteen of 20 studies evaluated the dimensional bone-level changes and bone regeneration using different methods (Table 5).

**Table 5:** Dimensional bone alteration and bone regeneration outcomes in the evaluated studies

Study	*n*	Test	Control	Method	Results test	Results control	Statistical significance
Castro et al. [41]	20	L-PRF	Spontaneous healing	CBCT (thickness buccal bone 1 mm below the crest) after 3 months	1.1 ± 0.3 mm	1.1 ± 0.4 mm	No
		A-PRF+			0.9 ± 0.3 mm		No
				CBCT (horizontal resorption 1 mm below the alveolar crest) after 3 months	Buccal: 1.6 ± 0.8 mm Palatal: 0.6 ± 0.7 mm	Buccal: 1,7 ± 1.0 mm Palatal: 0.5 ± 0.7 mm	No
					Buccal: 1.6 ± 0.7 mm Palatal: 0.7 ± 0.8 mm		No
				CBCT (horizontal resorption 3 mm below the alveolar crest) after 3 months	Buccal: 1.5 ± 0.8 mm Palatal: 0.4 ± 0.4 mm	Buccal: 1.4 ± 0.8 mm Palatal: 0.3 ± 0.4 mm	No
					Buccal: 1.2 ± 0.6 mm Palatal: 0.4 ± 0.7 mm		No
				CBCT (horizontal resorption 5 mm below the alveolar crest) after 3 months	Buccal: 1.0 ± 0.7 mm Palatal: 0.2 ± 0.4 mm	Buccal: 1.0 ± 0.6 mm Palatal: 0.1 ± 0.6 mm	No
					Buccal: 0.8 ± 0.6 mm Palatal: 0.3 ± 0.6 mm		No
				CBCT (socket fill) after 3 months	85.2 ± 22.9%	67.9 ± 19.2%	Yes: *p* = 0.005
					83.8 ± 18.4%		Yes: *p* = 0.01
				Histomorphometry (percent of bone volume/ tissue volume)	47.7 ± 7.9%	34.7 ± 6.9%	Yes: *p* < 0.05
					54.5 ± 5.6%		Yes: *p* < 0.05
				Micro-CT (percent of bone volume/ tissue volume)	43.4 ± 8.7%	35.0 ± 8.2%	No: *p* = 0.09
					50.7 ± 4.5%		Yes: *p* < 0.001
Sharma et al. [54]	30	PRF	Spontaneous healing	Digital panoramic radiographs (grayscale value) after 16 weeks	91.980	88.689	No: 0.668
Canellas et al. [47]	45	L-PRF	Spontaneous healing	CBCT (bone loss 1 mm below the alveolar crest) after 3 months	0.93 ± 0.9 mm	2.27 ± 1.2 mm	Yes *P* < 0.0001
				CBCT (bone loss 3 mm below the alveolar crest) after 3 months	0.85 ± 0.8 mm	1.67 ± 1.1 mm	Yes *p* < 0.005
				CBCT (bone loss at the buccal bone wall) after 3 months	0.70 ± 0.7 mm	1.39 ± 1.2 mm	Yes *P* < 0.02
				CBCT (horizontal bone loss at 5 mm below the crest) after 3 months	0.67 ± 0.5 mm	1.08 ± 1.0 mm	No *P* = 0.094
				CBCT (vertical bone loss at the palatal/ lingual wall) after 3 months	0.67 ± 0.9 mm	1.24 ± 1.15 mm	No *P* = 0.064
				CBCT (new bone formation) after 3 months	190.90 ± 169.90 mm^3^	44.87 ± 200.20 mm^3^	Yes *P* = 0.009
				Histomorphometry (percentage of new bone formation after 3 months)	55.96 ± 11.97%	39.69 ± 11.13%	Yes *p* = 0.00001
Srinivas et al. [48]	30	PRF	Spontaneous healing	CBCT (bone density)	24 h: 319.79 ± 95.472 3 months: 564.76 ± 94.856	24 h: 194.82 ± 78.986 3 months: 295.87 ± 87.217	24 h: Yes *P* < 0.001 3 moths: Yes *P* < 0.001
				CBCT (Bone hight)	24 h: 13.93 ± 3.56 mm 3 months: 12.28 ± 3.84 mm	24 h: 14.68 ± 4.32 mm 3 months: 12.78 ± 3.82 mm	24 h: No *P* = 0.466 3 moths: No *P* = 0.615
Ahmed et al. [53]	54	PRF	Spontaneous healing	Radiographic analysis (bone hight reduction, crest to tip of the root taking adjoining tooth as a guide) after 16 weeks	0.17 ± 0.44 mm	2.12 ± 0.69 mm	Yes *p* < 0.001
				Radiographic analysis (bone width reduction) after 16 weeks	0.47 ± 0.36 mm	1.71 ± 0.49 mm	Yes *p* < 0.001
				Radiographic analysis (bone density change using gray scale histogram) after 16 weeks	0.44 ± 1.21	1.45 ± 0.51	Yes *P* = 0.006
Areewong et al. [55]	38	PRF	Spontaneous healing	Histomorphometric: new bone formation ratio after 8 weeks	31.33 ± 18%	26.33 ± 19.63%	No *P* = 0.431
Zhang et al. [49]	28	PRF	Spontaneous healing	CBCT after 3 months (reduction of the buccal alveolar)	1.6000 ± 1.46416 mm	2.8000 ± 1.81487 mm	No
				CBCT after 3 months (reduction of the height of the lingual/palatal alveolar crest)	1.0000 ± 0.70711 mm	2.0500 ± 1.29180 mm	No
				CBCT after 3 months (reduction of the width of the alveolar crest)	1.0500 ± 0.77862 mm	2.0760 ± 1.67149 mm	No
				Histomorphometric analysis after 3 months (osteoid) area/tissue area)	9.7624 ± 4.0121%	2.8056 ± 1.2094%	Yes *P* < 0.01
Kumar et al. [43]	48	PRF	Spontaneous healing	Clinical measurement using measured using metal callipers (average loss of alveolar width) after 6 months	3 ± 0.8 mm	3.3 ± 0.61 mm	No
				Clinical measurement using measured using metal callipers (average loss of alveolar height) after 6 months	3 ± 0.64 mm	3 ± 0.83 mm	No
				Radiological measurement (degree of alveolar fill) after 6 months	73.76 ± 0.14%	74.3 ± 0.13%	No
Clark et al. [39]	45	Test 1: A-PRF Test 2: A-PRF + FDBA	Control1: spontaneous healing Control 2: FDBA	Clinical measurement using alginate impression and a periodontal probe (loss ridge height reduction) after 15 weeks	Test 1: A-PRF: 1.8 ± 2.1 mm Test 2: A-PRF + FDBA: 1.0 ± 2.3 mm	Control 1: 3.8 ± 2.0 mm Control 2: 2.2 ± 1.8 mm	Test 1 vs. control 1: Yes *P* < 0.05 Test 2 vs. control 2: No
				Clinical measurement using alginate impression and a metal callipers (Loss of ridge width (coronal)) after 15 weeks	Test 1: A-PRF: 2.8 ± 1.2 mm Test 2: A-PRF + FDBA: 1.9 ± 1.1 mm	Control 1: 2.9 ± 1.7 mm Control 2: 2.5 ± 1.1 mm	Test 1 vs. control 1: No Test 2 vs. control 2: No
				Clinical measurement using alginate impression and a metal callipers (Loss of ridge width (middle)) after 15 weeks	Test 1: A-PRF: 1.8 ± 1.8 mm Test 2: A-PRF + FDBA: 1.7 ± 1.2 mm	Control 1: 1.8 ± 1.3 mm Control 2: 1.5 ± 1.2 mm	Test 1 vs. control 1: No Test 2 vs. control 2: No
				Clinical measurement using alginate impression and a metal callipers (Loss of ridge width (apical)) after 15 weeks	Test 1: A-PRF: 1.8 ± 1.5 mm Test 2: A-PRF + FDBA: 1.6 ± 1.5 mm	Control 1: 1.5 ± 1.6 mm Control 2: 1.2 ± 1.3 mm	Test 1 vs. control 1: No Test 2 vs. control 2: No
				Micro-CT analysis of bone cores (bone mineral density) after 15 weeks	Test 1: A-PRF: 493 ± 70 mg/cm^3^ Test 2: A-PRF + FDBA: 521 ± 58 mg/cm^3^	Control 1:487 ± 64 mg/cm^3^ Control 2: 551 ± 58 mg/cm^3^	Test 1 vs. control 1: No Test 2 vs. control 2: No
				Histomorphometric analysis (percentage of vital bone) after 15 weeks	Test 1: A-PRF:46% ± 18% Test 2: A-PRF + FDBA: 29% ± 14%	Not reported	Test 1 vs. control 1: No Test 2 vs. control 2: No
Alzahrani et al. [44]	24	PRF	Spontaneous healing	Clinical Cast analysis (reduction of alveolar ridge width)	Week 1: 2.09 ± 0.84 mm Week 4: 5.22 ± 0.80 mm Week 8: 8.58 ± 1.73 mm	Week 1: 3.26 ± 2.21 mm Week 4: 9.79 ± 6.02 mm Week 8: 13.54 ± 6.57 mm	Week 1:No *P* = 0.141 Week 4:Yes *P* = 0.012 Week 8:Yes *P* = 0.036
				Radiolographic analysis (mean radiographic bone fill)	Week 1: 74.05 ± 1.66% Week 4: 81.54 ± 3.33% Week 8: 88.81 ± 1.53%	Week 1: 68.82 ± 1.07% Week 4: 74.03 ± 1.22% Week 8: 80.35 ± 2.61%	Week 1: Yes *P* = 0.012 Week 4:Yes *P* = 0.00 Week 8: Yes *P* = 0.017
Temmerman et al. [50]	22	PRF	Spontaneous healing	CBCT after 3 months (Vertical resorption, lingual)	0.4 ± 1.1 mm	0.7 ± 0.8 mm	No
				CBCT 3 months (Vertical resorption, buccal)	0.5 ± 2.3 mm	1.5 ± 1.3 mm	Yes *P* = 0.0002)
				CBCT 3 months (Horizontal width reduction 1 mm below the crest)	22.84 ± 24.28%	51.92 ± 40.31%	Yes *P* = 0.0004
				CBCT 3 months (Horizontal width reduction 3 mm below the crest)	6.16 ± 6.16%	14.51 ± 19.6%	Yes *p* = 0.007
				CBCT 3 months (Horizontal width reduction 5 mm below the crest)	2.91 ± 4.54%	4.4 ± 4.89%	Yes *P* = 0.02
				CBCT 3 months (socket fill)	8.1 ± 3.1 mm 94.7 ± 26.9%	6.2 ± 3.9 mm 63.3 ± 31.9%	Yes, *P* = 0.005 Yes, *P* = 0.0004
Suttapreyasri et al. [45]	8	PRF	Spontaneous healing	Clinical socket dimension measurements after 8 weeks (mesial-distal [M-D] and buccal-lingual [B-L]) were measured from the inner socket orifice at the midpoint of the extraction site, a UNC-15 periodontal probe (Hu-Friedy, Chicago, IL)	M-D: 1.76 ± 1.36 mm B-L: 3.31 ± 0.09 mm	M-D 2.17 ± 1.65 mm B-L: 3.92 ± 0.64 mm	M-D: no B-L: no
				Cast analysis buccal contour change after 8 weeks	Upper part: 1.96 ± 1.10 mm Middle part: 1.79 ± 0.90 mm Lower part: 0.5 ± 0.72 mm	Upper part: 2.59 ± 0.7 mm Middle part: 1.61 ± 0.43 mm Lower part: 0.56 ± 0.38 mm	Upper part: no Middle part: no Lower part: no
				Cast analysis lingual contour change after 8 weeks	Upper part: 1.59 ± 0.64 mm Middle part: 0.42 ± 0.39 mm Lower part: 1.78 ± 0.57 mm	Upper part: 1.03 ± 0.57 mm Middle part: 1.78 ± 0.47 mm Lower part: 0.39 ± 0.35 mm	Upper part: no Middle part: no Lower part: no
				Radiographic Resorption of Marginal Bone Levels at Mesial of the Extraction Site after 8 weeks	2.22 ± 0.51 mm	2.86 ± 0.65 mm	No
				Radiographic Resorption of Marginal Bone Levels at distal of the Extraction Site after 8 weeks	2.08 ± 0.09 mm	2.10 ± 0.50 mm	No
Hauser et al. [46]	23	PRF	Spontaneous healing	Clinical measurements of the alveolar crest Width loss after 8 weeks	0.48%	3.68%	Yes *P* < 0.05
				Radiographical analysis of the vertical linear Measurements on Superimposable Radiographs after 8 weeks	Mesial: 1.21 ± 0.4 mm Distal: 0.76 ± 0.25 mm	Mesial: 0.77 ± 0.17 mm Distal: 2.07 ± 0.81 mm	Mesial: yes Distal: no
				Micro CT (bone volume/Tissue Volume) after 8 weeks	0.281 ± 0.037	0.249 ± 0.037	No
Thakkar et al. [52]	36 sites	demineralized freeze‑dried bone allograft (DFDBA) mixed with PRF, covered by a collagen membrane	Demineralized freeze‑dried bone allograft (DFDBA), covered by a collagen membrane	calibrated radiographs (Ridge width reduction from baseline to 180 days)	0.75 ± 0.49 mm	1.3611 ± 0.70 mm	Yes (*p* = 0.005)
			Ridge height reduction (from baseline to 180 days)		1.38 ± 0.50 mm	1.08 ± 0.428 mm	No
Yewale et al. [51]		Sybograf plus (70% HA and 30% βTCP) mixed with A-PRF + , covered by a Collagen sponge (Collasponge ™)	Sybograf plus (70% HA and 30% βTCP), covered by a Collagen sponge (Collasponge ™)	CBCT after 6 months: horizontal width at 1 mm	2.123 ± 0.76 mm	1.83 ± 0.8 mm	No
				CBCT after 6 months: horizontal width at 3 mm	1.689 ± 0.84 mm	0.596 ± 1.08 mm	Yes (*p* = 0.041)
				CBCT after 6 months: horizontal width at 5 mm	0.97 ± 1.28 mm	0.59 ± 1.59 mm	No

*Clinical evaluation.* Kumar et al. [43] applied a clinical measurement method using metal capillaries to assess the width and height bone loss after 6 months. In both cases, no statistical significant differences were found between the PRF and the control group (spontaneous wound healing). Additionally, Clark et al. [39] evaluated the bone dimension change after an average of 15 weeks (3.75 months) using alginate impression and periodontal probe. The results showed that the A-PRF group underwent significantly lower ridge height reduction compared to the control group. However, no statistical significant differences were found when assessing the alveolar bone width. Alzahrani et al. [44] analyzed the alveolar ridge with reduction after 1, 4 and 8 weeks using cast analysis. The results showed statistically significantly lower reduction in the PRF group after 4 and 8 weeks (1 and 2 months) compared to the control group. Moreover, Suttapreyasri et al. [45] did not show any statistical significant difference in the alveolar bone width and buccal and lingual contour changes using cast analysis after 8 weeks. Hauser et al. [46] reported on statistically significantly lower percent of alveolar crest width resorption in the PRF group compared to the control group after 8 weeks (Table 5).

*Cone beam computer tomography. (CBCT)* CBCT measurements of the dimensional bone alteration after 3 months were performed by Canellas et al. [47] the results showed statistically significantly lower bone resorption in the PRF group compared to the control group especially in the 1–3 mm below the alveolar crest and the buccal wall. However, no difference was shown, when evaluating the horizontal bone loss. Additionally, the total volume of new bone formation was statistically significantly higher in the PRF group compared to the control group. Similarly, Srinivas et al. [48] showed statistically significantly higher bone density in the PRF group after 3 months by CBCT analysis. However, no differences were found in the bone height change. A further study by Zhang et al. [49] performed CBCT analysis to assess bone resorption after 3 months. They showed markedly lower resorption in all dimensions in the PRF group compared to the control group. However, they did not report statistical significant differences. Moreover, Temmerman et al. [50] analyzed the bone changes using CBCT and showed significantly lower vertical resorption in the PRF group compared to the control group, especially in the buccal wall. Similarly, a significantly lower horizontal bone resorption was shown in the 1–5 mm below the alveolar crest in the PRF group compared to the control group. The percent socket fill was significantly higher in the PRF group compared to the control group. Castro et al. [41] also demonstrated a significantly higher socket fill after 3 months using CBCT measurement in the PRF treated group compared to the untreated control (Table 5).

Clark et al. [39] also analyzed the dimensional ridge reduction after treatment using a bone substitute material in combination with PRF compared the treatment using the biomaterial alone. No statistical significant differences were shown in the clinical evaluation. Two further studies analyzed the socket augmentation using PRF in combination with bone substitute materials compared to the augmentation using native bone substitute material without PRF. Thakkar et al. [52] showed that the addition of PRF significantly reduce the ridge width reduction after 6 months. However, no significant difference was found when evaluating the ridge height reduction. Yewale et al. [51] showed significantly higher alveolar width preservation in the group of A-PRF + only when measured at 3 mm below the alveolar crest (Table 5).

*Two-dimensional X-ray evaluation.* Conventional radiologic analysis performed by Ahmed et al. [53] showed significantly lower resorption in the PRF group after 4 months compared to the control group. Kumar et al. [43] showed no statistical significant differences between the groups when considering the percent of socket fill after 6 months. By contrast Alzahrani et al. [44] used similar evaluation method and showed significantly higher percent of bone fill in the PRF group after 2 months compared to the control group. Suttapreyasri et al. [45] analyzed the resorption of marginal bone at the mesial and distal sites after 2 months and did not show statistically significant differences. Whereas Hauser et al. [46] performed similar measurements after 2 months and showed statistically significant differences, especially in the mesial site. Moreover, Sharma et al. [54] did not show statistical significant differences, when analyzing the bone density by means of gray scale after 16 weeks of healing (Table 5).

*Histologic evaluation.* Four of the 20 evaluated studies analyzed bone core biopsies by histology. Focus was placed on the evaluation of the percent of new bone formation by histomorphometry. Canellas et al. [47]; Zhang et al. [49] and Castro et al. [41] showed significantly higher percent of new bone formation in the PRF group after 3 months compared to the control group. Areewong et al. [55] (healing time: 8 weeks) and Clark et al. [39] (healing time 15 weeks) did not show statistical significant differences in the ratio of new bone formation (Table 5).

*Micro-computer tomography (micro-CT)* Clark et al. [39] and Hauser et al. [46] analyzed core biopsies using micro-CT. Bone volume to tissue volume analysis after 8 weeks did not show any differences between the PRF and control group. Similarly, the bone density measurement after 15 weeks did not show statistically significant differences. Castro et al. [41] showed a statistically significantly higher percent of bone volume/tissue volume when comparing the group of A-PRF + to the untreated control. However, no statistical significant differences were documented for the L-PRF group (Table 5).

### Secondary outcomes

The results of the secondary outcomes are described below.

#### Pain assessment

Six studies evaluated the patients pain reports using the visual analogue scale (VAS) by comparing socket treatment by means of PRF to spontaneous healing. 66.6% of the studies showed statistically significantly lower pain in the PRF group compared to the spontaneous wound healing [42, 50, 56, 57]. Ustaoglu et al. [42] showed that both L-PRF and T-PRF significantly reduced patients’ pain on day 1 compared to the control group of spontaneous wound healing. However, on day 2 the pain was reduced in both groups without statistical significant differences. Maurao et al. [56] showed that L-PRF significantly reduced patients pain on day 7 compared to the spontaneous healing without PRF. Kumar et al. [43] reported that 18% of the patients of the control group (spontaneous healing) reported on pain, whereas 0% of the PRF group had pain on day 1. Asmael et al. [58] did not show any statistical significant difference between the PRF treated side and the control side in their split mouth RCT. Tammerman et al. [50] evaluated patients pain on day 3 and showed that L-PRF significantly reduced the pain in comparison to the spontaneous wound healing. Additionally, Marenzi et al. [57] showed significant differences in the pain reduction of the L-PRF group compared to the spontaneous wound healing on early time point. However, the differences subsided on day 4, (Table 6).

**Table 6:** Pain assessment outcomes in the evaluated studies

Study	*n*	Test	Control	Results test	Results control	Statistics
Mourao et al. [56]	32	L-PRF	Spontaneous healing	7 days: 4 ± 1.15	7 days: 5.12 ± 1.08	Yes (*p* = 0.0128)
Ustaoglu et al. [42]	57	L-PRF	Spontaneous healing	Day 1: 3.30 ± 2.07 Day 2: 0.48 ± 0.92	Day 1:5.11 ± 1.60 Day 2	Day 1: yes (*P* = 0.047) Day 2: No
		T-PRF	Spontaneous healing	Day 1: 3.29 ± 1.85 Day 2: 0.47 ± 0.62		Day 1: yes (*P* = 0.047) Day 2: No
						T-PRF vs. L-PRF No
Kumar et al. [43]	48	PRF	Spontaneous healing	Day 1: 0% of the patients	Day: 1 18.1% of the patients	Not reported
Asmael et al. [58]	20	PRF	Spontaneous healing	48 h After Extraction: 0.65	48 h After Extraction: 1.8	No
Temmerman et al. [50]	22	L-PRF	Spontaneous healing	Day 3: 2,81	Day 3: 3,52	Yes *P* = 0.03
Marenzi et al. [57]	26	L-PRF	Spontaneous healing	3.2 ± 0.3	4.5 ± 0.7	Yes *P* < 0.0001
Yewale et al. [51]	20	Sybograf plus (70% HA and 30% βTCP) mixed with A-PRF + , covered by a Collagen sponge (Collasponge ™)	Sybograf plus (70% HA and 30% βTCP), covered by a Collagen sponge (Collasponge ™)	Pain frequency after 10 days: Mild:2 Moderate:8	Pain frequency after 10 days: Mild:3 Moderate: 7	No

One study by Yewale et al. [51] evaluated pain assessment after bone augmentation using bone substitute materials in combination with PRF versus bone substitute material alone (*n* = 10 per group). The results were not statistically significant.

#### Soft tissue regeneration

Soft tissue regeneration was evaluated in 8 studies, mainly using the soft tissue healing index by Landry et al. [59]. Two studies showed no statistically significant differences between the L-PRF, A-PRF + groups compared to the spontaneous wound healing after one week [40, 42]. Six studies (75% of the evaluated studies) reported remarkable improvement of the soft tissue healing in the L-PRF and T-PRF groups compared to the spontaneous wound healing, especially in the early healing time point of one week [48, 53, 54, 56, 57]. Additionally, Ustaoglu et al. [42] evaluated the percent of epithelialization and showed statistically significantly faster epithelization in the L-PRF and T-PRF groups compared to the spontaneous wound healing on both time points week 1 and 2, whereas Asmael et al. [58] did not record any statistical significant difference between the evaluated groups (Table 7).

**Table 7:** Soft tissue healing outcomes in the evaluated studies

Study	*n*	Test	Control	Method	Results test	Results control	Statistics
Sharma et al. [54]	30	PRF	Spontaneous healing	The Landry wound healing index (mean ± SD)	Day 3: 3.43 ± 0.504 Day 7: 3.93 ± 0.254 Day 14: 4.83 ± 0.379	Day 3: 3.17 ± 0.379 Day 7: 3.73 ± 0.082 Day 14: 4.3 ± 0.46	Day 3: yes *p* = 0.025 Day 7: yes *P* = 0.039 Day 14: yes *p* = 0.00
Mourao et al. [56]	32	L-PRF	Spontaneous healing	Wound healing index (mean ± SD)	Week 1: 3.81 ± 0.65 Week 2: 4.75 ± 0.44	Week 1: 3.18 ± 0.54 Week 2: 4.5 ± 0.51	Week 1: Yes *p* = 0.0138 Week 2: No
Srinivas et al. [48]	30	PRF	Spontaneous healing	Wound healing index after 7 days	3.8 ± 0.40	3.0 ± 0.53	Yes *P* < 0.001
Ahmed et al. [53]			Spontaneous healing	Wound healing index	Very good 94.1%	Very good in 86.7%	Not reported
Ustaoglu et al. [42]	57	L-PRF	Spontaneous healing	The Landry wound healing index (mean ± SD)	Week 1: 3.58 ± 0.63 Week 2: 4.59 ± 0.51	Week 1: 3.21 ± 0.66 Week 2: 4.38 ± 0.49	Week 1: no Week 2: no
			Spontaneous healing	Complete wound epithelization (%)	Week 1: 54.9 Week 2: 100	Week 1: 10.1 Week 2: 40.7	Week 1: yes *P* = 0.047 Week 2: yes *P* = 0.041
		T-PRF	Spontaneous healing	The Landry wound healing index (mean ± SD)	Week 1: 3.69 ± 0.51 Week 2: 4.71 ± 0.50	Week 1: 3.21 ± 0.66 Week 2: 4.38 ± 0.49	Week 1: no Week 2: no
			Spontaneous healing	Complete wound epithelization (%)	Week 1: 70.1 Week 2: 100	Week 1: 10.1 Week 2: 40.7	Week 1: yes *P* = 0.047 Week 2: yes *P* = 0.041
Giudice et al. [40]	40	A-PRF +	Spontaneous healing	Wound healing index (mean)	Week 1: 1 Week 2: 0.25	Week 1: 1.05 Week 2: 0.33	No
		L-PRF	Spontaneous healing	Wound healing index (mean)	Week 1: 0.95 Week 2: 0.15	Week 1: 1.05 Week 2: 0.33	No
Asmael et al. [58]	20	PRF	Spontaneous healing	Percentage of epithelization after 1 week	52.7%	51.3%	No
				The Landry wound healing index (mean) after 1 week	3.45	4.2	Yes *P* = 0.0035
15 Marenzi et al. [57]		L-PRF	Spontaneous healing	Wound healing index (mean ± SD)	Day 3: 4.8 ± 0.6 Day 7: 4.5 ± 0.5 Day 14: 4.2 ± 0.2 Day 21: 4.1 ± 0.1	Day 3: 5.1 ± 0.9 Day 7: 4.9 ± 0.3 Day 14: 4.3 ± 0.3 Day 21: 4.2 ± 0.2	Day 3: No *p* = 0.197 Day 7: yes *p* = 0.05 Day 14: yes *p* = 0.01 Day 21: yes *p* = 0.0002

## Discussion

Blood concentrates and especially PRF gained increasing interest in the oral and regenerative medicine in the last decade [31]. PRF is applied for different indications to support wound healing and regeneration of both bone and soft tissue. Recently, several systematic reviews evaluated the existing clinical evidence of PRF in different fields including oral and maxillofacial surgery [33, 60, 61] and orthopedics [62]. However, most of recent reviews analyzed more than one indication and used a broad set of inclusion criteria, which hardly allow drawing concise conclusions for specific indications of PRF [33, 60, 61]. Additionally, focus was frequently placed on the general bone regeneration only, as an important parameter for implantology, whereas little is known about the influence of PRF on specific parameters of wound healing including soft tissue regeneration and pain. Interestingly, these factors were shown to contribute to patients satisfaction and the long-term success of dental implants. Additionally, many studies did not use “reasonable” control groups thus involving several additional cofactors [61, 63]. For example several studies were conducted to compare PRF in the test group with a collagen-based biomaterials [64, 65] or mineralized bone substitute materials [64, 66] as a control group. In this context, it has to be noted that PRF is an autologous bioactive blood concentrate system based on the blood components including platelets and leukocytes, that are embedded in a fibrin network [21]. It does not exhibit the physicochemical characteristics of conventional biomaterials [21, 23]. Therefore, it is not comparable to other biomaterials such as bone substitute materials or collagen-based membranes. Accordingly, a precise control group is needed to evaluate the efficacy of PRF in the regeneration process. Hence, in this systematic review the native blood clot as a process of the spontaneous wound healing was considered as the most suitable and reasonable control group to assess the regenerative potential and efficacy of PRF. If biomaterials were utilized in combination with PRF, they had to be identical in the test and control groups. This restriction to reasonable control groups additionally aimed to exclude bias from additional cofactors potentially influencing the regeneration process. Based on this hypothesis, the present review addressed the following focused question: in patients with freshly extracted teeth, what is the efficacy of PRF in in the prevention of pain and the regeneration of soft tissue and bone compared to the respective control without PRF treatment?

The literature research revealed only 20 studies eligible for the evaluation. In total 17 studies (RCTs and CCTs) analyzed the effect of PRF compared to the spontaneous wound healing. One study included four groups and evaluated the treatment of PRF alone in the first test group compared to the spontaneous wound healing and in the second group the combination of PRF with a bone substitute material in the second test group compared to the bone substitute material alone in the second control group. Only 2 studies evaluated the combination of PRF with bone substitute materials in comparison to bone substitute material without PRF.

A relatively high bias risk was assessed for most of the studies, especially concerning blinding of patients and outcome assessment. Another limitation is the report on the morphology of the treated defects, i.e., the anatomy of the socket after tooth extraction in terms of the presence, quality and dimension of the buccal wall as well as the status of bone resorption at the time point of tooth extraction. Recent studies showed that among others these parameters are highly important for the progress of the regeneration process after tooth extraction and may predefine the risk of bone atrophy [5, 6]. These limitations in the data acquisition point to the necessity to improve the quality of reporting in future studies.

Additionally, when evaluating PRF it is important to analyze the preparation protocol. PRF is not a ready-to-use product, but a freshly prepared blood derivate for each individual patient. Recently, many different centrifugation protocols were reported in the literature [21, 29, 67, 68]. Additionally, there was a confusion in the literature concerning the reported parameters and the preparation methods [69, 70]. Recent studies explored the role of the centrifugation process in the preparation of PRF [67, 71–77]. These studies have shown that the applied RCF has a crucial influence on the components and the bioactivity of PRF, thus influencing its therapeutic efficacy [67, 71–77]. Thereby, the application of a high RCF during the centrifugation of PRF results in a significantly lower number of platelets, leukocytes and growth factor concentrations compared to PRF-matrices that are prepared using a low RCF [67, 71–77]. This phenomenon was proved in many studies and defined as the low-speed centrifugation concept (LSCC), which explained for the first time the role of the applied RCF in the preparation of blood concentrates [67]. In this context, three parameters are mainly important when reporting on the preparation of blood concentrates (a) the programmed revolutions per minutes (rpm), which is a parameter that appears on the centrifuge in most types and is usually adjustable; (b) the applied centrifugal force (RCF), a parameter that is mostly not visible on the centrifuge but can be calculated according to the centrifuge radius and (c) the centrifugation time. Moreover, the used tube surface also influences the quality and bioactivity of the resulted PRF [78].

Most of the studies evaluated in the present review reported only the applied rpm, without any information about the radius of the used centrifuge or the resulted RCF. Fourteen of the studies referred to the first introduced protocol referred to “L-PRF” or “Choukrouns PRF” and used a relatively high rpm of 2700–3000 for 10 to 12 min. Only three studies compared different PRF protocols including advanced PRF, that implements a medium RCF (1300 rpm, 208×*g*) or T-PRF, that implements specific titanium-based blood tubes. At this point, it has to be emphasized that the use of different preparation protocols results in different PRF-qualities that may manipulate the clinical outcome. Thereby, scientific reporting on PRF should include the above-mentioned parameters. Accordingly, the authors recommend a recently published guideline to report on the preparation of blood concentrates to be able to reproduce and evaluate the scientific data [78, 79].

Within the limitations of the acquired data, 66% of the evaluated studies showed that the application of PRF significantly reduced the postoperative pain, especially in early time points 1–3 days after surgery (Table 4). This observation may be explained by the autologous and bioactive character of PRF and the release of different growth factors and cytokines involved in pain control. The application of PRF provides the wound with all needed components to immediately start the healing process without the need for recruiting the immune cells to the injury area.

Additionally, 75% of the studies, that evaluated the influence of PRF on the soft tissue healing, showed that PRF promoted a significantly faster wound healing compared to the control group (Table 7). In this context, according to the wound healing index by Landry et al. [59], wound closure parameters were significantly better in the PRF group especially after 1 week of application. This finding reflects that PRF may be considered as an autologous wound healing booster to accelerate wound healing. Various studies have shown that PRF releases important growth factors such as epidermal growth factor (EGF), that promotes epithelialization, transforming growth factor beta (TGF-β), which is highly needed for fibroblasts proliferation and migration as well as vascular endothelial growth factor (VEGF), which is a key signal for neovascularization [67, 71–77].

The here reported clinical observations are in accordance with different preclinical studies showing the role of PRF in wound healing. In vitro studies used soft tissue regeneration model by combining fibroblasts and endothelial cells previously provided explanation on the possible mechanisms of PRF in promoting wound healing [74]. It was shown that in addition to the fibrin network, which provides a favorable scaffold for residual cells such as endothelial cells and fibroblasts, PRF serves as a drug delivery system by gradually releasing growth factors and promote the building of a well-defined vascular network as well as enhancing fibroblasts proliferation and migration [74, 77, 80]. Interestingly, the evaluated clinical studies reported mostly no significant difference in soft tissue healing after 2 weeks. This observation is logic, as the wound healing process under physiological conditions normally finalize after 2 weeks so that no differences between the evaluated groups are observed after this time period [81].

The analysis of the collected data concerning the efficacy of PRF in bone regeneration showed different outcomes according to the evaluation time point and applied method. Eleven studies reported on bone regeneration outcomes. Most of them evaluated bone regeneration after 8 to 15 weeks. Three of four studies reporting on clinical measurements showed significantly lower bone resorption in the PRF group compared to the control group, especially when considering the buccal wall and the ridge height. Similarly, CBCT evaluation of bone resorption, bone density and socket fill showed significantly lower resorption in the PRF group compared to the control group after 8–15 weeks. Especially, the 1–3 mm below the alveolar crest were well preserved in the PRF group compared to the control group. Interestingly, one study reported on bone regeneration after 6 months using clinical measurements and did not show any differences between the PRF and control group.

Within the limitations of these data, a very important finding may be highlighted by this analysis concerning the most suitable time point for implant insertion after socket preservation using PRF. Based on the present results, it seems that PRF promotes accelerated soft tissue and bone regeneration within the early healing phase. Apparently, PRF is effective in delaying bone resorption, but it cannot prevent it on the long run. Thereafter, the effect of PRF subsided, so that no difference could be observed after 6 months. These findings appear to be plausible when looking at the properties of PRF, which is an autologous, bioactive, fibrin-based scaffold, and different from ready-to-use biomaterials with stable scaffolds such as collagen-based biomaterials or bone substitute materials [67, 71–77]. An in vivo study has shown that PRF degrades after 2–3 weeks, which is a sufficient time period to expand its effect on the early wound healing [23]. By contrast, the degradation time periods of conventional biomaterials such as collagen matrices or bone substitute materials ranged from 3 months to years according to the biomaterial specific characterization [16]. Therefore, when working with PRF, it is important to understand its characteristics as a fibrin-based scaffold and not as classical biomaterials.

Thereby, the present systematic review suggests considering PRF as a further group of regenerative biomaterials called blood-concentrates in addition to the xenogeneic, allogeneic and synthetic biomaterials. This specific group of blood concentrates provided completely different benefits and requirements and may be considered as an adjuvant therapy [82]. Accordingly, different treatment protocols apply for blood concentrates and they should not be treated likewise to the classical biomaterials in terms of guided bone regeneration (GBR) and guided tissue regeneration (GTR). Classical GBR/GTR biomaterials are inactive acellular materials, that require sufficient time until integrating into the implantation bed and allowing for cell migration and therefore initiation of the regeneration process [83, 84]. Therefore, more time is needed in this case until the socket is ready for implantation. However, PRF is as a bioactive scaffold including crucial blood cells that are necessary for the regeneration process and can accelerate the phases of wound healing and starts the regeneration process earlier.

The physiological atrophy process after tooth extraction was described as a rapid and continuous process. About 50% of the alveolar bone atrophies in the first 3 months after tooth extraction [7, 85]. Especially, in the first 3 months after tooth extraction the efficacy of PRF in delaying bone resorption was evidenced in the here evaluated studies. Consequently, after a period of 6 months the effect of PRF subsided and bone atrophy as described earlier. Only two studies were found, that evaluated the combination of bone substitute materials with PRF in comparison to the native bone substitute material without PRF. Based on the small number of patients and the limited data, it is not possible to draw a conclusion concerning the efficacy of PRF when combined with biomaterials. Therefore, further well-designed RCTs are needed to answer this question.

None of the here evaluated studies reported on the efficacy of PRF to reduce scar formation during soft tissue healing. Although liquid PRF is applied in esthetic treatment for skin rejuvenation and scar treatment [86, 87]. Additionally, a recent study reported on the efficacy of PRF in promoting wound healing in large defects after three-dimensional augmentations in terms of the open healing concept as an alternative to flap mobilization and to avoid flap dehiscence [3]. Moreover, no data were found about the implant survival rate of implants placed in sockets treated with PRF compared to the spontaneous wound healing. Eventually, none of the evaluated studies reported on any adverse or server reactions related to the application of PRF.

Altogether, the analysis of the available evidence of 20 prospective, controlled studies highlighter the efficacy of PRF in supporting socket healing after tooth extraction. PRF was demonstrated to promote soft tissue regeneration, to reduce the postoperative pain and preventing bone dimensional bone loss in the early period of 2–3 months. This evidence refers to PRF protocols using a high RCF during the preparation. It has to be stated that the number of available studies in this field is very limited, and that the risk of bias was high. Future studies are needed to evaluate further PRF protocols using a lower RCF protocols to further investigate the potential benefit of different preparation protocols as an indication-specific approach.

## Conclusion

The present reviews aimed to provide clinical evidence on the efficacy of PRF in the treatment of fresh extraction sockets in comparison to the spontaneous wound healing. Within the limitations of the collected data, PRF was found to be effective in reducing post-operative pain, accelerating soft tissue healing and preventing bone dimensional bone loss, especially in the early time period of 2–3 months. Although the present review focused only on prospective randomized controlled and controlled studies, a relatively high risk of bias was assessed, especially in the categories blinding of participant and personnel as well as blinding of outcome assessment. Additionally, the here evaluated data showed a high heterogeneity in the used methods for outcome measures. Therefore, it was not possible to perform a meta-analysis.

## Data Availability

Not applicable.

## Abbreviations

BSMs: Bone substitute materials
PRP: Platelet-rich plasma
PRGF: Plasma-rich in growth factors
PRF: Platelet-rich fibrin
L-PRF: Leukocytes-rich platelet-rich fibrin
CCT: Controlled clinical trial
RCTs: Randomized controlled clinical trial
RPM: Round per minute
RCF: Relative centrifugal force
VAS: Visual analogue scale
CBCT: Cone beam computer tomography
Micro-C: Micro-computer tomography
GBR: Guided bone regeneration
GTR: Guided tissue regeneration
